# Endothelin-Converting Enzyme 1 and Vascular Endothelial Growth Factor as Potential Biomarkers during *Ex Vivo* Lung Perfusion with Prolonged Hypothermic Lung-Sparing

**DOI:** 10.1155/2022/6412238

**Published:** 2022-02-07

**Authors:** Claudia Hernández-Jiménez, J. Raúl Olmos-Zúñiga, Matilde Baltazares-Lipp, Rogelio Jasso-Victoria, Adrián Polo-Jerez, María Teresa Pérez-López, Luis Florentino Vázquez-Justiniano, Néstor Emmanuel Díaz-Martínez, Miguel Gaxiola-Gaxiola, Laura Romero-Romero, Axel Edmundo Guzmán-Cedillo, Mario Enrique Baltazares-Lipp, Juan Carlos Vázquez-Minero, Luis Horacio Gutiérrez-González, Marcelino Alonso-Gómez, Mariana Silva-Martínez

**Affiliations:** ^1^Department of Surgical Research, National Institute of Respiratory Diseases Ismael Cosío Villegas, Mexico City, Mexico; ^2^Experimental Lung Transplant Unit, National Institute of Respiratory Diseases Ismael Cosío Villegas, Mexico City, Mexico; ^3^Nursing Research Coordination, National Institute of Respiratory Diseases Ismael Cosío Villegas, Mexico City, Mexico; ^4^Laboratory of Cellular Reprogramming and Tissue Engineering, Department of Medical and Pharmaceutical Biotechnology, Center for Research and Assistance in Technology and Design of the State of Jalisco, A.C, Mexico City, Mexico; ^5^Laboratory of Morphology, National Institute of Respiratory Diseases Ismael Cosío Villegas, Mexico City, Mexico; ^6^Department of Pathology, School of Veterinary Medicine and Zootechnics, UNAM, Mexico City, Mexico; ^7^Hemodynamics and Echocardiography Service, National Institute of Respiratory Diseases Ismael Cosío Villegas, Mexico City, Mexico; ^8^Cardiothoracic Surgery Service, National Institute of Respiratory Diseases Ismael Cosío Villegas, Mexico City, Mexico; ^9^Department of Virology and Mycology, National Institute of Respiratory Diseases Ismael Cosío Villegas, Mexico City, Mexico

## Abstract

Lung transplantation requires optimization of donor's organ use through ex vivo lung perfusion (EVLP) to avoid primary graft dysfunction. Biomarkers can aid in organ selection by providing early evidence of suboptimal lungs during EVLP and thus avoid high-risk transplantations. However, predictive biomarkers of pulmonary graft function such as endothelin-converting enzyme (ECE-1) and vascular endothelial growth factor (VEGF) have not been described under EVLP with standard prolonged hypothermic preservation, which are relevant in situations where lung procurement is difficult or far from the transplantation site. Therefore, this study is aimed at quantifying ECE-1 and VEGF, as well as determining their association with hemodynamic, gasometric, and mechanical ventilatory parameters in a swine model of EVLP with standard prolonged hypothermic preservation. Using a protocol with either immediate (I-) or delayed (D-) initiation of EVLP, ECE-1 levels over time were found to remain constant in both study groups (*p* > 0.05 RM-ANOVA), while the VEGF protein was higher after prolonged preservation, but it decreased throughout EVLP (*p* > 0.05 RM-ANOVA). Likewise, hemodynamic, gasometric, mechanical ventilatory, and histological parameters had a tendency to better results after 12 hours of hypothermic preservation in the delayed infusion group.

## 1. Introduction

Lung transplantation (LTx) is the last resort treatment for patients with severe chronic lung disease. Unfortunately, only approximately 15% of donor lungs are suitable for transplantation, the remainder being generally inadequate due to brain death-induced lung injury and ICU-related complications, resulting in endothelial dysfunction and edema formation [1]. Therefore, LTx requires optimal utilization of available donors and the optimization of donor's organ use. This has led to the emergence of ex vivo lung perfusion (EVLP) as a tool for evaluation, preservation, and reconditioning of the donor's lung prior to transplantation [2], since it allows the evaluation of lungs under continuous physiological monitoring, reconditioning lungs with fluid removal, and intervention/engineering of lungs with intense therapy during extended preservation. However, it can also induce inflammation, compromise cellular metabolism and mitochondrial function, alter microcirculation, and cause ventilation-induced lung injury [3]. During EVLP, hemodynamic criteria, pulmonary mechanics, and gas exchange are important in the decision to accept a graft. With these parameters, approximately 20% of donor lungs perfused in EVLP for 4 to 6 hours are rejected for clinical transplantation due to poor physiologic performance. In addition, there is also a small percentage of cases that, despite having favorable physiology during EVLP, develop primary graft dysfunction (PGD) after transplantation [4]. PGD pathogeny involves multiple pathways such as inflammation, innate immunity, platelet dysfunction for coagulation, and fibrinolysis that may cause endothelial and epithelial lesions in the lung. Endothelial dysfunction is manifested by the activation of endothelial biomarkers such as endothelin (ET) and vascular endothelial growth factor (VEGF), which could lead to reduced graft survival after brain death. In these circumstances, biomarkers can aid in organ selection by providing early evidence of suboptimal lungs during EVLP and thus avoid high-risk transplantations [4, 5]. Endothelin-converting enzyme (ECE-1) is essential for the synthesis of endothelin (ET), which is a 21 amino acid family of peptides and exists in three isoforms: ET-1, ET-2, and ET-3. ET-1 is the most abundant isoform, which acts as a potent vasoconstrictor, smooth muscle cell and fibroblast mitogen, and a stimulator of inflammatory cell infiltration [6–8]. Moreover, ET-1 increases the expression of cell adhesion molecules, indicating a link between ET-1 and endothelial dysfunction which mediates increased permeability and edema in the lungs; this can be used as a predictor of PGD and bronchiolitis obliterans [9]. Given that VEGF is the major regulator of vascular permeability, ET-1 can promote VEGF expression in lung endothelial and epithelial cells [10]. Nevertheless, its overexpression may aggravate present edema.

Currently, applying the EVLP platform, it has been demonstrated that lung viability can be successfully maintained despite a prolonged period of cold preservation [11–13]. This may be especially relevant in situations or places where lung procurement is difficult or far from the transplantation site (a frequent problem in developing countries) so that the graft has to be maintained at hypothermic conditions for prolonged periods. However, predictive biomarkers of pulmonary graft function such as ECE-1 (the ET-1 precursor) and VEGF have not been described under cold preservation conditions. Therefore, this study is aimed at quantifying ECE-1 and VEGF, as well as determining their association with hemodynamic, gasometric, and mechanical ventilatory parameters, in a porcine model of EVLP with standard prolonged hypothermic preservation.

## 2. Materials and Methods

### 2.1. Experimental Animals

This study was made at the Department of Surgical Research of the National Institute of Respiratory Diseases Ismael Cosío Villegas (INER). Ten healthy domestic swine, regardless of sex, weighing between 18 and 20 kg were used. This protocol was reviewed and approved by the Bioethics Committee of the INER (IRB B25-13). All animals were treated in strict accordance with the Technical Specifications for the Care and Use of Laboratory Animals of the Mexican Official Standard NOM-062-ZOO-1999 and the Guide for the Care and Use of Laboratory Animals [14, 15]. The sample size was reduced in agreement with the principles of experimental techniques proposed by Balls and Kilkenny et al. [16, 17].

### 2.2. Study Groups

All animals underwent cardiopulmonary block procurement and were divided as follows:

Group I (*n* = 5): the immediate EVLP (I-EVLP) group underwent lung procurement and normothermic EVLP.

Group II (*n* = 5): delayed initiation of EVLP (D-EVLP) period of prolonged standard hypothermic preservation (12 hours) in Perfadex® solution (XVIVO Göteborg, Sweden) and subsequent EVLP.

The lungs of all swine were perfused ex vivo for a continuous 4-hour period, during which the parameters of lung function were assessed as described below.

### 2.3. Anesthesia and Surgical Procedure

All procedures were performed under general anesthesia. Induction was performed with tiletamine-zolazepam (4 mg/kg, IM. Zoletil, Virbac, Carros, France) and propofol (4 mg/kg, IV. Recofol, PISA, Guadalajara, JAL, Mexico), then maintained with isoflurane (Forane, Abbott Mexico S.A. de C.V., Mexico City, Mexico) and fentanyl (0.1 mg/kg, IV. Fentanest, Janssen-Cilag, Puebla, Mexico) as analgesic. The animals were ventilated with pulmonary protection strategies. Subsequently, cardiopulmonary block procurement was performed with the technique described by Mariscal et al. [18].

### 2.4. Lung Preparation for EVLP

**Figure 1:**
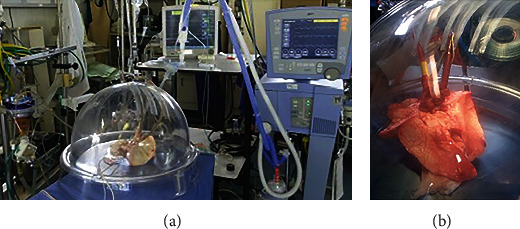
(a) Ex vivo lung perfusion (EVLP). The EVLP system is composed of a pump, ventilator, heating unit, deoxygenator, perfusate reservoir, and organ dome. (b) The cannulated pulmonary artery, left atrium, and intubated trachea in the organ dome.

EVLP was performed as described previously by Cypel et al. [19]. In brief, a funnel-shaped cannula (Vitrolife, Göteborg, Sweden) was sewn to the left atrial cuff, a cannula (Vitrolife, Göteborg, Sweden) was secured into the pulmonary artery (PA), and a 7.0-8.0 endotracheal tube with the balloon removed was secured into the trachea. The EVLP circuit consisted of extracorporeal circulation with a neonatal reservoir VHK 1100 (Maquet Getinge Group, Germany) and a neonatal oxygenator Quadrox-i (Maquet Getinge Group, Germany) connected to a pump CDL-10140 (Gambro, USA). The lungs were transferred to an XVIVO chamber (XVIVO Göteborg, Sweden), and retrograde flow was initiated through the left atrium to de-air the pulmonary vasculature and flush any remaining clot. The PA cannula was then connected, and antegrade flow was begun at 0.1 L/min. EVLP was performed using acellular Steen solution (XVIVO Göteborg, Sweden), a commercially available preservative solution designed for ex vivo lung assessment, supplemented with 10,000 IU heparin (APP Pharmaceuticals, Schaumburg, Ill, USA). The perfusate was slowly warmed to 37°C during 30 minutes as the flow was titrated up to the target of 40% of the estimated cardiac output (100 mL/kg). When the perfusate reached 32°C, ventilation was initiated with room air at a tidal volume of 6-8 mL/kg, respiratory rate of 8 breaths/min, and positive end-expiratory pressure (PEEP) of 5.0 cm H2O. Recruitment maneuvers are performed every hour to a pulmonary artery wedge pressure (PawP) of 25 cm H_2_O [18]. After initiation of ventilation, a mixture of 6% oxygen, 8% carbon dioxide, and 86% nitrogen were infused into the membrane oxygenator to deoxygenate the PA perfusate and allow for accurate measurement of lung oxygenation capability (Figure 1). Every hour after EVLP initiation, PaO_2_ was evaluated with fraction of inspired oxygen (FiO_2_) at 21%, and after, the lungs were ventilated with (FiO_2_) at 100% for 10 minutes, and another sample of the perfusate was taken from the left atrial return for gas analysis [12, 19].

### 2.5. Lung Physiology Assessment

The study was conducted for 4 hours. The hemodynamic, gasometric, and ventilation mechanics parameters were assessed: cardiac output was determined using the thermodilution method (Hemodynamic Profile CARESCAPE B650 (General Electric Company©, Finland)), pulmonary vascular resistance (PVR), partial pressure of oxygen (PaO2), and partial pressure of carbon dioxide (PaCO_2_) were measured every hour (ABL 800 Flex Analyzer (Radiometer, Brønshøj, Denmark)); static (Cstat) and dynamic (Cdyn) lung compliance, airway resistance (Raw), and peak inspiratory pressure (PIP) were measured every hour (Avea™ VIASYSTM Healthcare, USA).

### 2.6. Histological Assessment of the Lungs

Open lung biopsy (OLB) was performed to obtain pulmonary specimens suitable for histologic analysis. Biopsies were taken at the beginning and every hour during the EVLP; samples were obtained from right lobes in all experiments with areas of the lung whose macroscopic appearance presented lesions, trying to cover the transition areas between sites with areas of normal appearance. Biopsy samples were stained with hematoxylin and eosin (H&E) and analyzed for pathological changes. The most prominent features observed in the lungs were used to develop a scoring system: evidence of cell infiltration into the lungs (neutrophils, macrophages, and lymphocytes), presence of edema, and formation of alveolar injury. The severity of the findings was graded on a scale from 0 (absent) to 3 (severe) [20].

### 2.7. Radiography

Radiographic images of the lungs were taken before and hourly during EVLP. All radiographs were taken in the anterior-posterior plane in the supine position. Opacity was quantified according to their extension and reported on a scale of 0 to 4: no opacity (grade 0), opacity up to 25% (grade 1), 26 to 50% (grade 2), 51 to 75% (grade 3), and 76 to 100% (grade 4). The score was determined in a single-blind analysis [21].

### 2.8. Determination of Pulmonary Edema

Pulmonary edema was quantified by gravimetric analysis. The lung tissues were weighed and dried in an oven between 60 and 65°C up to constant weight. Finally, the lung weight gain was calculated with the following formula: ΔPP = (PH − PS)/PS, where *Δ*PP is lung weight gain, PH is final lung weight, and PS is initial lung weight.

### 2.9. Cytokine and Oxidative Damage Levels

Cytokine and oxidative damage levels were quantified in triplicate at the beginning and the end of EVLP in bronchoalveolar lavage (BAL). An enzyme-linked immunosorbent assay (ELISA) was used to determine tumor necrosis factor alpha (TNF-*α*) (Thermo Fisher Scientific KSC3011, Waltham, MA, USA), IL-8 (Invitrogen™ KSC0081, Vienna, Austria), and porcine protein carbonyl (E07P0048 CBP kit, CBP BlueGene Biotech, CO, LTD Shanghai, China).

### 2.10. Western-Blot Analysis

Total protein concentrations in tissue lysates were measured by Lowry assay. Samples of the protein (150 *μ*g/ml) were then separated by using 10% sodium dodecyl sulfate-polyacrylamide gel electrophoresis and transferred onto nitrocellulose transfer membranes (Bio-Rad Laboratories, Inc. USA). The membranes were then probed by using antibodies against ECE-1 (Cat. 6855b, ABGENT, San Diego, CA, USA), dilution: 1 : 1000 in 0.1% BSA in PBS and VEGF (Cat. 115544, Biorbyt, Germany; 1 : 500 in milk 4%). Immune complexes were detected with goat anti-rabbit IgG HRP (Cat. HAF008, R&D Systems, USA; 1 : 1000 BSA 4%). The blots were then visualized by using chemiluminescence (ChemiDoc™ XRS + System, Bio-Rad Laboratories, Inc. USA), and the signal intensity was quantified by densitometry using Image Lab™ Software (Bio-Rad Laboratories, Inc. USA).

### 2.11. Data Analysis

Statistical analysis of parametric data was done with repeated measures (RM -ANOVA). Post hoc comparisons at specific time points were evaluated using the Bonferroni significant differences test. The nonparametric Mann–Whitney test or Wilcoxon signed-rank test was used to compare statistical difference between two groups and Friedman's two-way analysis of variance by ranks of related samples. The Shapiro Wilk for the sample distribution and Student's *T*-test for comparison of group means were used. SPSS 19.0 statistical software (SPSS Inc., Chicago, USA) was used, and *p* values of *p* < 0.05 were considered significant.

## 3. Results

All blocks completed the four hours of ex vivo lung perfusion. All parameters were within normal values for pigs.

### 3.1. Gas Exchange

**Figure 2:**
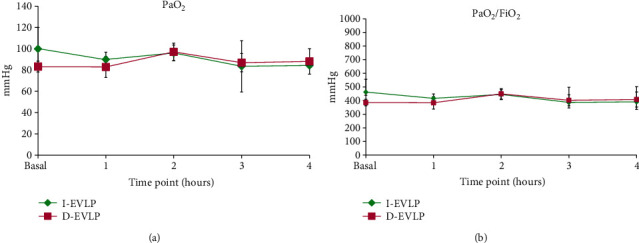
PaO_2_ levels and PaO_2_/FiO_2_ ratio. Although there is a trend of higher values in the D-EVLP group, especially at 2 and 4 hours, no significant differences are found. The lungs of the D-EVLP group mainly reached higher values at each time point and throughout the course of EVLP than the lungs of the I-EVLP group. (Mean ± SD, *p* > 0.05 RM-ANOVA).

PO_2_ remained above 80 mmHg over time, with FiO_2_ at 21% (*p* = 0.189). Oxygenation did not differ between I-EVLP and delayed EVLP (D-EVLP) (*p* = 0.551) (Figure 2(a)). PaCO_2_ I-EVLP (19.58 ± 6.99) and D-EVLP (25.20 ± 5.58) levels did not vary over time (*p* = 0.964), nor amongst groups (*p* = 0.551). Likewise, the PaO_2_/FiO_2_ ratio (I-EVLP 390.06 ± 112.56, D-EVLP 407.77 ± 55.45) showed no difference over time (*p* = 0.551) nor between groups (*p* = 0.964) (Figure 2(b)).

### 3.2. Functional Outcomes

**Figure 3:**
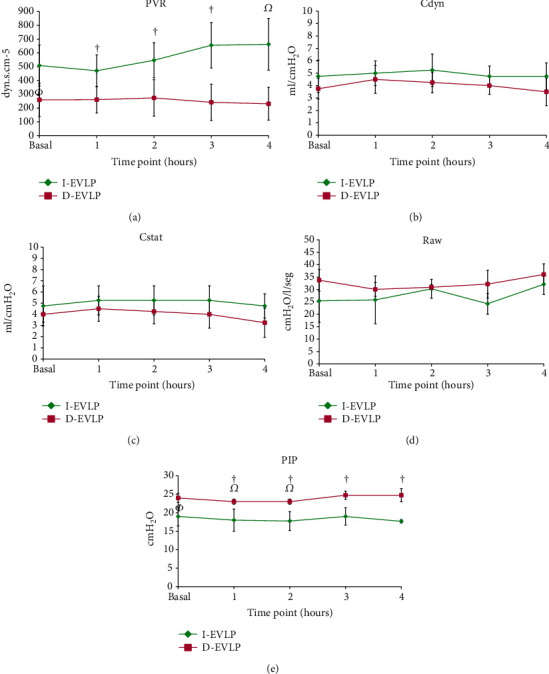
Functional results of immediate (I-EVLP) and delayed (D-EVLP). Values are expressed as mean ± SD, RM-ANOVA Bonferroni. (a) PVR towards higher values in the I-EVLP group; (b) and (c) dynamic compliance decreased over time and lowered in the D-EVLP group; (d) and (e) both groups showed almost constant values for Raw and PIP, which were generally higher in the D-EVLP group during the entire process (*^Φ^p* < 0.05 between groups, ^†^*p* < 0.05 between groups at the same time, and *^Ω^p* < 0.05 compared with basal I-EVLP).

For immediate EVLP (I-EVLP), PVR was increased at 2 hours and continued with that trend until the end of EVLP, with significant difference over time (*p* = 0.010) and when comparing between groups (*p* = 0.007) (Figure 3(a)).

During EVLP, static (Cstat) and dynamic (Cdyn) compliance changed over time, with a slight decrease at 4 hours for the D-EVLP group and no significant differences between groups at any other time point for both groups (Cstat (*p* = 0.190) and Cdyn (*p* = 0.187)) (Figures 3(b) and 3(c)).

Airway resistance (Raw) was increased in both study groups, but there was no difference between them within any time point (*p* = 0.067) (Figure 3(d)). The PIP in both study groups was maintained close to its basal levels, however, the comparison between groups I-EVLP (*p* = 0.000) was significantly lower with differences between groups at all-time points (*p* = 0.0001) (Figure 3(e)).

### 3.3. Radiologic Assessment

In both groups, slight edema was present (*p* > 0.05 Mann–Whitney *U*-test); nevertheless, there was a time frame in the I-EVLP group in the left lung after two hours with moderate edema, while the other time frame of the same group presents severe edema in both lungs at the end of the study (*p* > 0.05 Friedman two-way). In contrast, one group of the D-EVLP during a time frame showed moderate edema in both lungs in the basal radiography, after one and two hours, it remained present only in the right lung (*p* > 0.05 Friedman two-way).

### 3.4. Histologic Findings

Individual lung injury severity score parameters showed a better assessment in the D-EVLP group compared to the I-EVLP group (*p* > 0.05 Mann–Whitney *U*) with lower presence of neutrophils, macrophages, and lymphocytes; the D-EVLP group also had less alveolar edema, still, these variables did not reach statistical significance (*p* > 0.05 Friedman's two-way).

### 3.5. Gravimetric Findings

A trend towards lower levels of wet-dry ratio in the D-EVLP group compared to the I-EVLP group was observed after 4 h of EVLP, with no significant differences (*p* = 0.998).

### 3.6. Measured Cytokines

Both groups had significantly higher levels of IL-18 in comparison with its baseline, I-EVLP (*p* = 0.06) and D-EVLP (*p* = 0.10), and there were no significant differences between groups (*p* = 0.97). However, I-EVLP levels were higher in the I-EVLP group (998.61 ± 293.60) compared with D-EVLP (982.55 ± 301.53). Likewise, TNF*α* differed according to its respective baseline values for D-EVLP (*p* = 0.043) and I-EVLP (*p* = 0.043), with higher values for I-EVLP (327.448 ± 107.94) and D-EVLP (272.08 ± 167.32), without significant differences between groups (*p* = 0.548).

In the analysis of the protein carbonylation data, normality was found (*p* > 0.05 Shapiro-Wilk). In group, I-EVLP had a slight increase at the end of the process (*p* = 0.562) in contrast to group D-EVLP, which presented a decrease (*p* = 0.844). When both groups were compared, no significant difference was found (*p* = 0.772).

### 3.7. Western Blot Analysis

**Figure 4:**
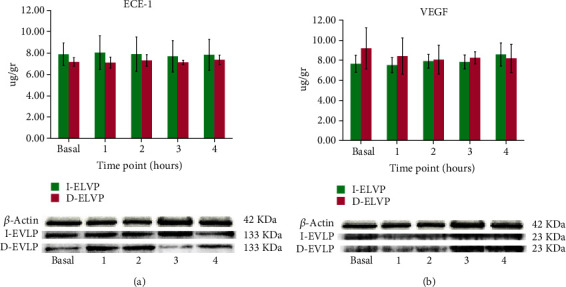
Western blots of ECE and VEGF in the lungs of I-EVLP and D-EVLP. (a) Densitometry showing relative levels of ECE-1 and (b) VEGF. Mean ± SD (*p* > 0.05).

ECE-1 and VEGF levels were similar in both groups. There was no significant difference between groups, nor when comparing each time frame of the study with its respective baseline (*p* = 0.444, *p* = 0.722) for ECE-1 and VEGF, respectively (Figures 4(a) and 4(b)).

## 4. Discussion

Twelve hours of exposure before EVLP did not negatively affect in a significant way neither the proteins ECE-1 and VEGF nor pulmonary function. The evaluation of I-EVLP and D-EVLP groups was similar; even though I-EVLP presented a rise in the wet-dry weight ratio and lightly augmented histological findings, and oxygenation capacity (partial oxygen pressure at FiO_2_ of 100%) in both groups was maintained at above-expected values for EVLP lungs [22, 23]. Our data matched other studies that found that lungs with D-EVLP maintain pulmonary function in a significant way [12] and showed in a porcine model that EVLP with prior cold static preservation for nine hours is as safe and effective as I-EVLP of donor's lungs procurement [13].

In our study, both groups showed a hemodynamically similar performance during the ex vivo evaluation, although the PVR was higher for the I-EVLP group from the initial measurement, suggesting the persistence of sparse microthrombi that, despite the use of perfadex solution and heparin, caused flow obstruction at the level of small arterioles; subsequently, the trend of increasing PVR over time suggests endothelial dysfunction with inflammation of endothelial cells leading to decreased capillary diameter probably due to injury by ischemia-reperfusion (IR) [24, 25]. Accordingly, our histological findings showed increasing numbers of inflammatory cells in our I-EVLP group in comparison with the D-EVLP group.

Compliance of lung tissue was comparable in both groups, but a trend to higher Cstat and Cdyn in the I-EVLP group was observed. The slight compliance decrease in D-EVLP may be due to longer hypothermia, which would slow the process of reaching the same lung elasticity as in the I-EVLP group [13]. The PIP remained at the same level in both groups during EVLP, with higher values in D-EVLP; however, in porcine models, PIP is higher compared to human lungs [13], and although there was a significant difference between groups, the ∆ peak-plateau was not greater than 5 cm H_2_O. Airway and vascular pressures remained stable for 4 h. All other measured lung functional parameters, in both groups, did not differ. Additionally, the water content of the lung tissue confirmed by a higher wet-dry ratio and histology in group I-EVLP, it can be interpreted as damage caused by a certain degree of damage to the alveolar-capillary barrier due to thinning and rupture caused by lungs ischemia [26]. IL-8 has been usually associated to acute lung injury, as it can be a biomarker; for PGD cases, with values significantly higher at both 1 and 4 hours of EVLP [4]. It also been known that elevated IL-8 and mRNA before TNF-*α* implantation correlates with mortality after 30 days of lung transplant [27]. In our work, the increase of cytokines levels is likely related to IR, as it is an inflammatory response that involves injury/dysfunction of endothelium and epithelium, with activation of molecular patterns associated with damage. The ex vivo lung continues to be an important part of generation of strong inflammatory response, as it harbors leukocytes in its alveolar and interstitial compartments [5].

The lower levels of cytokine releases in the D-EVLP group may be associated with hypothermic preservation-induced-ischemia [25, 28], in agreement with those obtained in lungs in hypothermic preservation for 4 h with retarded EVLP [12, 13]. However, cytokine reduction in lung perfusate did not affect oxygenation, PVR, or edema formation, demonstrating that other factors play a significant role in graft dysfunction [29].

The D-EVLP group showed decreased oxidative stress that may be associated with hypothermic preservation [30]. Although protein carbonylation in porcine EVLP had not been reported, the carbonyl content of BAL fluid proteins has been found to increase in ARDS patients [31]. The rapid formation of protein carbonyl groups during protein oxidation may favor its use as biomarker, in a time-frame of hours and days instead of minutes, as usual with lipid peroxidation products that are degraded in minutes [32].

Even though increased levels of ET-1 are associated with PGD, in approximately 30% of lung transplant cases [7, 8], the role of ECE-1 and VEGF in conditions of prolonged hypothermia and EVLP has not been previously described. Thus, the results of this work may be clinically relevant, and a positive stimulation of ECE-1 would lead to an increase in ET-1. ECE-1 levels remain constant over time in both study groups, in agreement with a clinical trial of EVLP in which increased levels of ET-1 and Big ET-1 were associated with lungs declined for transplantation and those that developed PGD [8]. However, our data did not come from brain (BDD) and cardiac (DCD) dead donor's lungs, which may represent differences in protocols [33, 34].

In this work, the VEGF protein levels decreased throughout D-EVLP, in contrast with the I-EVLP group, in which it increased at the end of EVLP. This can be explained by VEGF overexpression in several cell types (such as activated alveolar epithelial type 2, endothelial cells), which increases the vascular permeability. Also, VEGF increases in response to oxygen radicals and cytokines, involved in ischemia-induced lung injury [35]. Our results on the W/D ratio in the D-EVLP group revealed less edema formation, which is consistent with other works that found reduced W/D ratio and pulmonary neutrophil infiltration in a VEGF treatment group compared with a LPS group [36]. On the other hand, upregulated expression of protein levels VEGF-A and -C, but not -B, as well as increased tissue fluid contents in donor grafts versus controls has been found, suggesting that the molecular permeability pathway described here for the VEGF family might be of benefit to selectively counteract edema formation in lung grafts [35]. Moreover, serum VEGF levels measured preoperatively after hospital admission were higher in recipients who developed PGD than in recipients who did not, suggesting that preexisting vascular endothelial injury is a risk factor for development of PGD [37].

One limitation of this work is the fact that the lungs were not transplanted at this stage of the study, in addition to having a small number of samples; additionally, the levels of Big endothelin and ET were not determined. However, both protocols indicate that after EVLP in the pretransplant stage, the ECE-1 and VEGF expression levels had changes that were not significantly affected. Likewise, comparable values were achieved in the hemodynamic, gasometric, mechanical ventilatory, and histological parameters with a tendency to better results after 12 hours of hypothermic preservation in the delayed infusion group.

## Data Availability

The data used to support the findings of this study are included within the article.

## Acronyms

Raw: Airway resistance
BDD: Brain-Dead Donors
BAL: Bronchoalveolar lavage
D-EVLP: Delayed initiation of EVLP
DCD: Donation after circulatory death
Cdyn: Dynamic lung compliance
ECE-1: Endothelin-converting enzyme
ELISA: Enzyme-linked immunosorbent assay
EVLP: Ex vivo lung perfusion
FiO_2_: Fraction of inspired oxygen
H&E: Hematoxylin and eosin
I-EVLP: Immediate EVLP
IR: Ischemia-reperfusion
LTx: Lung transplantation
OLB: Open lung biopsy
PaCO_2_: Partial pressure of carbon dioxide
PaO_2_: Partial pressure of oxygen
PIP: Peak inspiratory pressure
CBP: Porcine protein carbonyl
PEEP: Positive end-expiratory pressure
PGD: Primary graft dysfunction
PA: Pulmonary artery
PVR: Pulmonary vascular resistance
RM–ANOVA: Repeated measures
Cstat: Static lung compliance
TNF-*α*: Tumor necrosis factor alpha
VEGF: Vascular endothelial growth factor
